# Anatomical Insights: Two Case Reports on Protostylid Variants and Literature Synthesis

**DOI:** 10.7759/cureus.74480

**Published:** 2024-11-26

**Authors:** V Anu, T Manigandan, Xavier Dhayananth, T Sarumathi, T Vasanthakumar

**Affiliations:** 1 Research and Development, Bharath Institute of Higher Education and Research, Chennai, IND; 2 Public Health Dentistry, Sathyabama Dental College and Hospital, Sathyabama Institute of Science and Technology, Chennai, IND; 3 Oral Medicine and Radiology, Sree Balaji Dental College and Hospital, Chennai, IND; 4 Orthodontics and Dentofacial Orthopedics, Sathyabama Dental College and hospital, Sathyabama Institute of Science and Technology, Chennai, IND

**Keywords:** anomaly, dental caries, paramolar tubercle, prevention, primary dental care, variant

## Abstract

Protostylids are an anatomical variant of the paramolar tubercle, which refers to the presence of an additional cusp in the buccal surfaces of maxillary and mandibular bicuspids and molars. This structure, first reported by Dahlberg in 1950, is found in low frequencies and plays a significant role in dental anthropology. This anatomical variant, if present poses a threat to dental caries and periodontal diseases and hence requires early diagnosis and preventive dental care. Two cases of protostylids that were reported during dental consultations at a primary health care center in Semmencheri, Chennai are presented in this article with an attempt to educate on the presence of such trait so that dental professionals can identify and plan preventive dental care at the earliest.

## Introduction

The crowns of both primary and permanent dentition exhibit a wide morphological variation. One such morphological variant is the increase in the number of cusps called supernumerary cusps. These supernumerary cusps can occur either as a dental anomaly or as a normal anatomical variant [1].

Paramolar tubercle is an anatomical variant that refers to the presence of an additional cusp in the buccal surfaces of maxillary and mandibular primary molars and permanent maxillary and mandibular bicuspids and molars. Dahlberg referred to this structure as "parastyle" when it occurs in maxillary molars and "protostylid" when it is found in mandibular molars [2]. The anatomical variants occur in low frequencies and play a significant role in dental anthropology. Dahlberg was the first to report this supernumerary cusp on the deciduous molars of an Eskimo skull [3].

Chowdhry et al. report a high prevalence of 57.85% of protostylids among the population in the National Capital Region, India [4]. Data regarding its prevalence in other populations are limited to case reports [5-9]. This indicates that this tubercle might be underreported or left undiagnosed by dental professionals. Case reports available in the literature include a cusp with free apex.

Here, we describe two cases of protostylid that we came across during our dental consultation at a primary health care center in Semmencheri, Chennai.

## Case presentation

Case 1

**Figure 1:**
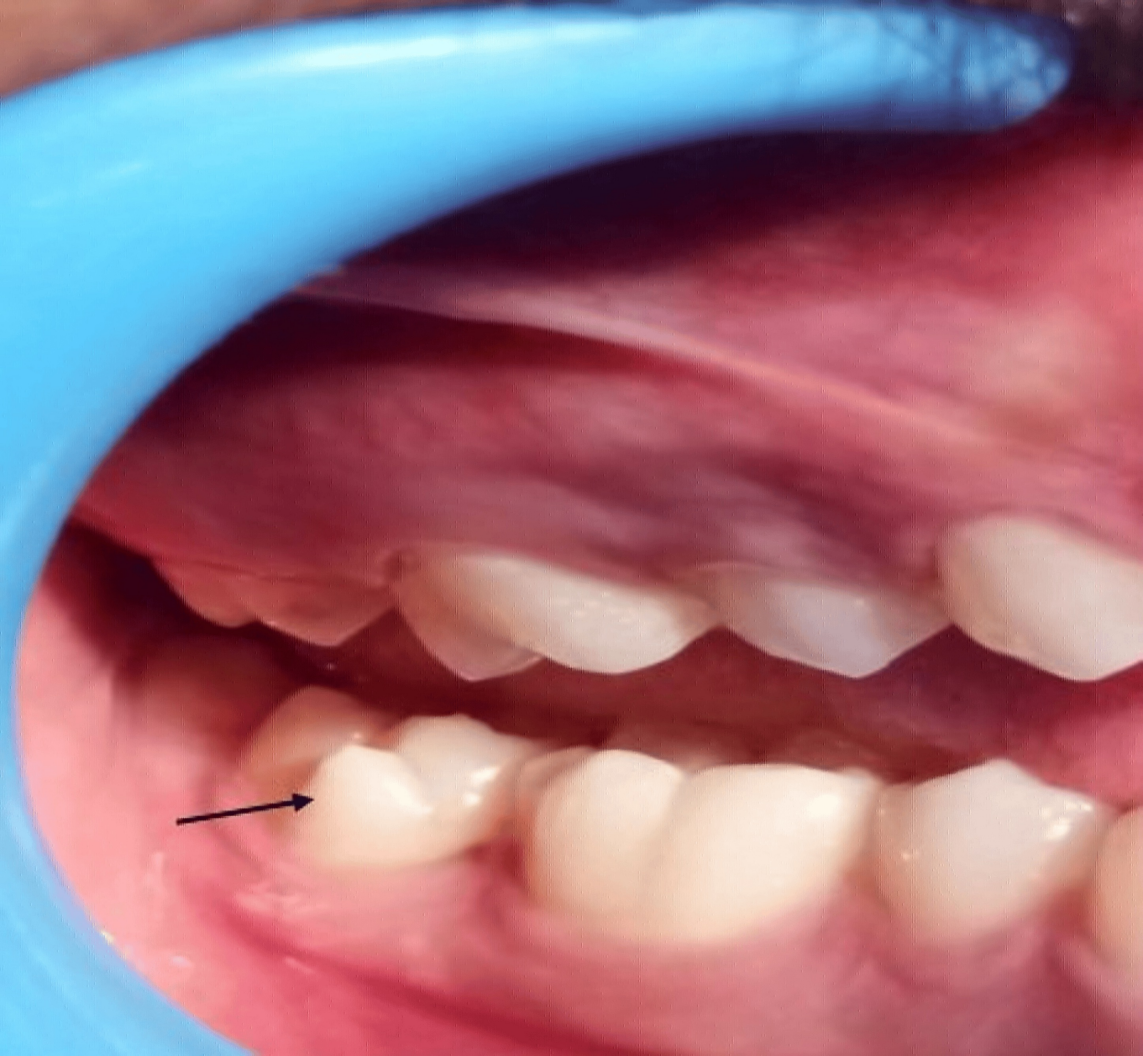
Protostylid present in the buccal surface of right second mandibular molar (case 1).

A male patient aged 18 years reported to the dental clinic at the outpatient department of the primary health center, Semmencheri, Chennai, with the chief complaint of pain in his upper right back tooth region for the past two weeks (Figure 1). There was no relevant past medical history. On clinical examination, the extraoral appeared normal. Intra-orally, there was an accessory cusp on the buccal surface of the permanent right mandibular second molar (47). The cusp measured 6 mm in height and width 8 mm at the base and 4 mm at the apex. The base of the cusp was located along the cervical margin of the tooth. The apex was located 4 mm below the occlusal plane. A developmental groove was present separating the apex of the cusp, indicating Score 7 (a cusp with a free apex occurs) according to the Arizona State University Dental Anthropology System (ASUDAS) grading [4]. The base of the cusps was along the gingival margin. There was no dental caries or gingival inflammation associated with the tooth. The soft tissue was normal and there was no other dental anomaly present.

Case 2

**Figure 2:**
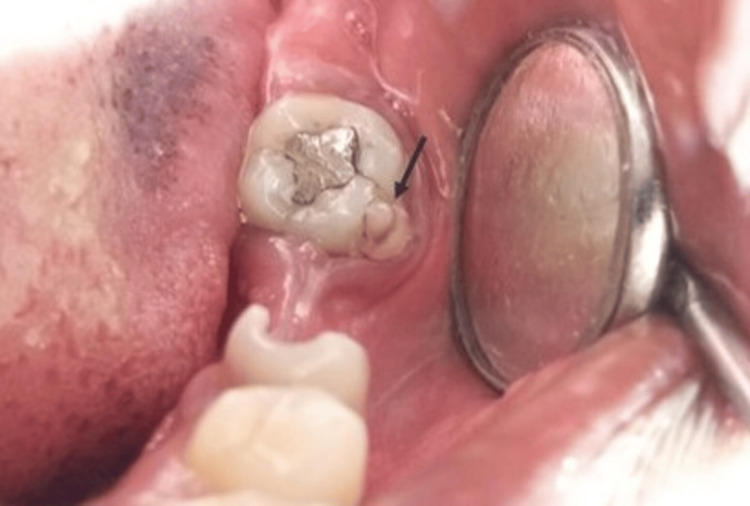
Protostylid present in the buccal surface of left second mandibular molar (case 2).

A female patient aged 35 years, reported to the outpatient department at the primary health center, Semmencheri, Chennai, with the chief complaint of pain in her lower right back tooth region for the past two weeks (Figure 2). There was no relevant medical or dental history. On clinical examination, extra orally there were no significant changes. Intraoral examination showed the presence of an accessory cusp on the mesiobuccal cusp of the left permanent mandibular second molar. The cusp measured about 3 mm in height from the base and width of 6 mm cervically and 3 mm along the apex. The base of the cusp was located along the cervical margin of the tooth and the apex was located 2 mm below the occlusal plane. A developmental groove was seen separating the cusp from the tooth surface which indicates Grade 7 (a cusp with a free apex occurs) according to the ASUDAS grading. There was a stained pit and fissure and no tooth wear. The gingiva showed signs of inflammation with bleeding on probing. Dental plaque and subgingival calculus were present along the left permanent mandibular second molar. All third molars were missing congenitally.

## Discussion

Dental cusps begin to develop during the early bell stage of tooth development. The inner enamel epithelial cells secrete certain activators and inhibitors that regulate the formation of cusp. The primary enamel knot is produced by the activators which get neutralized by the inhibitor when the concentration reaches threshold. Certain genes present in the cells code and control the activator and inhibitor expression and modulate the deposition of enamel. During amelogenesis, the primary knot forms the occlusal table of the bicuspids and molars while the secondary enamel knot forms the cusps [1,4].

Turner and Harris suggest that the paramolar tubercle, namely protostylid and parastyle would have arisen from an accessory enamel knot that developed at the surface where the apex forms during morphogenesis of tooth [1]. Gaspersic conducted a study in the crowns of 26 extracted human third molars that had protostylid to find out the microscopic characteristics of the enamel surface and structure of the protostylid [10]. He observed that the protostylids had varying sizes of grooves with the bottom exhibiting one or more pits of different sizes. He also observed the presence of a small cusp form at the amelodentinal junction below the pits, thus indicating that these cusps are formed during the morphogenetic phase.

Protostylids occur in the buccal surfaces of mandibular molars. It can be seen as a pit and distal bending of the buccal groove, or as a surface irregularity with varying degrees of prominence. These protostylids should not be considered a dental anomaly, as they are a derivative of the cingulum and thus represent morphological dental crown variation [8]. We have reported two cases of protostylid in permanent molars. The literature search shows the Indian case reports, summarized in Table 1. It could be noted that most of the reported cases have a free apex separated from the buccal surface of the lower molar by a developmental groove.

**Table 1:** Protostylid cases reported in India. BS: buccal surface, DB: distobuccal surface, MB: mesiobuccal surface, CEJ: cementoenamel junction, NR: not reported.

S. No	Author name/year of publication	State	Age/gender of patient	Paramolar tubercle reported site	Unilateral or bilateral	Appearance		Any groove present	Dental caries associated with the tubercle	Any other findings
						Base	Apex			
1	Appadurai et al. (2018) [7]	Tamil Nadu	30 years/female	Protostylid BS of 37	unilateral	NR	NR	NR	NR	Radiographic findings showed the cusp was devoid of any pulpal extension
2	Narsapur et al. (2017) [8]	Karnataka	28 years/female	DB of 36 and DB of 46	Bilateral	36 and 46 blends with cervical portion	Flat-free apex	Yes	Yes	Retained 53, missing 12. Peg lateral in 22, all third molars are missing
3	Desai et al. (2016) [11]	Jaipur	21 years/male	MB of 48	Bilateral	Below CEJ	3mm below the occlusal plane	NR	No	Frictional keratosis on the retromolar trigone area. Dental calculus was present along the tooth, inflamed gingiva
				Distal aspect of 38		NR	NR	No	No	Dental calculus was present along the tooth, inflamed gingiva
4	Shigli et al. (2010) [12]	Madhya Pradesh	14 years/female	MB of 46 and 36	Bilateral	NR	NR	Yes	Yes	Dental calculus was present along the tooth, inflamed gingiva

Classification of protostylid

The significance of paramolar tubercles is increasingly recognized by researchers, as they offer a valuable means of connecting this morphological trait to geographic distribution [11]. Earlier before 1920, all dental morphological characteristics were recorded based on the presence or absence of dichotomy of the trait. It was Ales Hrdlicka (1920) who first reported different grading systems as the morphological trait when present may show different forms. Dahlberg in the 1940s, developed a grading system for different morphological traits including protostylid. The Dental Anthropology Laboratory of Arizona State University developed standards for recording these morphological traits to increase the reliability of observers. This standard grading system is the Arizona State University Dental Anthropology System (ASUDAS) [4].

The standard system of grading protostylid according to ASUDAS is (i) grade 0: no expression of any sort, buccal surface is smooth; (ii) grade 1: a pit occurs in the buccal groove; (iii) grade 2: buccal groove is curved distally; (iv) grade 3: a faint secondary groove extends mesially from the buccal groove; (v) grade 4: secondary groove is slightly more pronounced; (vi) grade 5: secondary groove is stronger and can be easily seen; (vii) grade 6: secondary groove extends across most of the buccal surface of cusp 1, which is considered a weak or small cusp; and (viii) grade 7: a cusp with a free apex occurs.

Clinical implications and recommendations

The presence of protostylids can be a threat to major dental public health problems like dental caries and periodontal disease. The pits and groove might accumulate cariogenic bacteria leading to dental caries. The surface irregularity affects normal brushing leading to the development of dental plaque and dental calculus resulting in inflammation of the gingiva as seen in Case 2 and reports by Desai et al. and Shigli et al. [11,12]. If the grooves that separate the paramolar tubercle from the tooth extend into the root surface, it might cause vertical bone loss [9].

The protostylids are present in the buccal surface of mandibular molars and hence might interfere with occlusion if the apex is at or above the occlusal plane and might lead to occlusal disharmony such as habitual jaw repositioning. The apex of the cusp might cause irritation of the buccal mucosa leading to frictional keratosis [7].

The presence of additional cusps might interfere with the placement and adaptation of molar bands during orthodontic bands, indicating the need for its removal by ameloplasty [7]. It might also get affected if present in the abutment tooth during tooth preparation for crown prosthesis. In such cases, if the pulpal tissues are found to be present root canal therapy should be done before any other treatment with utmost care as there might be an additional root canal.

 These paramolar tubercles are extremely rare and hence can be used as an individual's unique identification [9].

## Conclusions

This report describes two cases of protostylid with Grade 7 ASUDAS (cusps with free apex). Protostylids are a morphological trait that is found to be rare or underreported. The presence of this creates morbidity of the tooth as well as habitual repositioning of the jaw. Early diagnosis and performing early preventive procedures directed to this would avoid the development of dental caries and periodontal diseases. The diagnosis of this tubercle can be missed by dental professionals due to their lack of exposure to the presence of this trait. This report exhorts dental professionals to become familiar with the morphological characteristics of teeth that occur unusually and report the cases. A population-based prevalence study can also be designed to know the frequency and variability of paramolar tubercles among different populations.
